# Effects of Gamma Irradiation on Inhibition of Urease Activity and Fishy Smell in Mackerel (*Scomber japonicus*) during Refrigerated Storage

**DOI:** 10.4014/jmb.2112.12037

**Published:** 2022-05-13

**Authors:** So-Mi Jeong, Han-Ho Kim, Si-Hyeong Ryu, Woo-Sin Kang, Ji-Eun Lee, Su-Ryong Kim, Ga-Hye Lee, Xiaotong Xu, Eui-baek Byun, Dong-Hyun Ahn

**Affiliations:** 1 Institute of Fisheries Sciences, Pukyong National University, Busan 46041, Republic of Korea; 2 Department of Food Science & Technology and Institute of Food Science, Pukyong National University, Busan 48513, Republic of Korea; 3 Advanced Radiation Technology Institute, Korea Atomic Energy Research Institute, Jeongup 34057, Republic of Korea

**Keywords:** Gamma irradiation, *Scomber japonicas*, fishy smell, *Vibrio parahaemolyticus*, urease

## Abstract

In this study, gamma-irradiated mackerel (*Scomber japonicus*) meat was stored in a refrigerator for 20 days to examine the physicochemical changes related to fishy smell. The effect of gamma irradiation on the inhibition of the activity of crude urease extracted from *Vibrio parahaemolyticus* was also evaluated. Increased levels of trimethylamine (TMA) and volatile basic nitrogen (VBN) content, which are the main components causing fishy smell, were significantly reduced by day 20 of storage after gamma irradiation, indicating that freshness was maintained during storage. The ammonia nitrogen contents of 3, 7, 10, and 20 kGy gamma-irradiated groups were significantly decreased by 6.5, 15.2, 17.4, and 23.9%, respectively, compared to non-irradiated groups on day 20 of storage. In addition, urease activity decreased in a gamma irradiation intensity-dependent manner. Volatile organic compounds (VOCs) were measured during the storage of gamma-irradiated mackerel meat. The contents of ethanol, 2-butanone, 3-methylbutanal, and trans-2-pentenal, which are known to cause off-flavors due to spoilage of fish, were significantly reduced by day 20 of storage. Therefore, gamma irradiation can be considered useful for inhibiting urease activity and reducing fishy smell during fish storage.

## Introduction

Recently, there has been an increasing demand for fish, as an excellent nutrient source rich in minerals such as selenium, and nitrogen compounds, including taurine and betaine, and vitamins [1]. However, fishy smell, induced by the deterioration of freshness after capture, is a major limiting factor in the consumption of fish, including the direct consumption and development of processed foods. Deterioration of fish is largely caused by autologous enzymes, microorganisms, and oxidation, while the substances that cause fishy smell due to freshness deterioration include trimethylamine (TMA), formaldehyde, hydrogen sulfide, methyl mercaptan, ammonia, and volatile basic nitrogen (VBN) [2-4].

Elasmobranchs, such as rays and sharks, contain large amounts of urea in their muscles, and urease secreted by microorganisms decomposes urea to produce ammonia. In the case of fish, urease in muscles makes the pH alkaline after death and volatility increases, resulting in a strong ammonia-like smell [5, 6]. *Vibrio parahaemolyticus* is a basophilic bacterium distributed in seawater and various seafood including fish and shellfish. *V. parahaemolyticus* is greatly affected by water temperature, salt concentration, pH, and levels of available organic matter. The species also produces various metabolites [7]. While several species of bacteria are reported to produce urease, about 8%of *V. parahaemolyticus* strains isolated from the marine environment were found to be urease-positive [8]. Therefore, to control the substances causing the fishy smell of fish and shellfish, it is important to inhibit the activity of enzymes that play important roles in the generation of the odor. Methods to suppress the fishy smell of fish mainly involve antioxidant action by adding various natural substances such as citron, coffee gourd, onion, ginger, *Zanthoxylum piperitum*, persimmon leaves, *Agastachis Herba*, rosemary, and sage extract [9-14]. However, so far, there has been a lack of research regarding the inhibition of the enzymes that generate fishy smells.

Mackerel (*Scomber japonicus*) contains large amounts of n-3 fatty acids, such as eicosapentaenoic acid, 20:5n-3 (EPA), docosahexaenoic acid, 22:6n-3 (DHA), polyunsaturated fatty acids (PUFA), and peptide and protein hydrolysates [15-17]. In addition, mackerel is a red meat fish with a very high lipid content, and it is known that spoilage proceeds faster in foods high in lipids than in other high-protein foods because there are many non-protein nitrogen components in the muscles, which are used by bacteria while the fish is decomposing [18]. In the case of red meat fish such as mackerel, secure storage stability is important because of high fatty acid content, which causes fishy off-flavors due to fatty acid rancidity during processing, and adversely affects product quality by promoting protein denaturation and lowering nutritional value [19]. Therefore, to resolve problems that occur in the distribution of red meat fish products such as mackerel, research is being actively conducted to improve their storage properties.

In previous studies to improve the storability of mackerel, non-heating treatments using ultraviolet (UV) irradiation, low-temperature osmotic dehydration, and high hydrostatic pressure (HHP) treatment have been performed [20-23].

Gamma irradiation is a representative example of recently used non-thermal processing technology with high potential for application. Gamma rays are an ionizing form of radiation that generates ions in atoms, atomic groups, and molecules when penetrating substances. Owing to its strong penetrating power, gamma irradiation is used for improving food properties, inhibiting the growth of microorganisms and viruses, and long-term preservation of food resources [24-25]. In addition, this technology has no residual toxicity and can maintain the original quality of food, thereby expanding its range of applications in food processing [26].

Therefore, in this study, we confirmed the beneficial effect of using gamma irradiation technology to inhibit urease activity, improve storability, and reduce the fishy smell of mackerel during refrigerated storage.

## Materials and Methods

### Mackerel Sample Preparation

Mackerel was purchased from a market in Busan, Korea. The bones, intestines, head, and skin were removed, and after the addition of 0.05% ascorbic acid, the meat was ground. The ground mackerel meat samples were then vacuum-packed for subsequent gamma irradiation.

### Preparation of Crude Urease from *V. parahaemolyticus*

Crude urease was extracted from *V. parahaemolyticus* isolated from *Saccharina japonica* collected in Busan, Songjeong, Korea. To estimate urease production in *V. parahaemolyticus*, Bacto urea broth and Christensen's urea agar (Difco, USA) were used. Luria Bertani medium (pH 5.5) supplemented with 2% NaCl, 0.5% glucose, and 0.2% urea was used as the culture medium for *V. parahaemolyticus*. Bacterial growth was examined by measuring optical absorbance at 600 nm using a spectrophotometer.

Crude urease was prepared according to the method of Kim and Kim [27]. After adding 20-30 ml of the pre-culture solution to 1 L of culture medium, incubation was performed at 37°C for 6-7 h, followed by centrifugation at 6,992 ×*g* for 10 min at 4°C. The precipitate was washed twice with saline solution and suspended in 20 mM phosphate buffer (pH 7.0). The suspension was crushed using an ultrasonic crusher (POWERSONIC 420, Hwashin Technology Co., Ltd., Korea) before being centrifuged (15,729 ×*g* for 10 min at 4°C) for use as a crude urease preparation.

### Gamma Irradiation

The vacuum-packed mackerel meat and crude urease samples were γ-irradiated at doses of 3, 7, 10, and 20 kGy. Gamma irradiation was performed using a cobalt-60 irradiator (point source AECL, IR-79; MDS Nordion International Co., Ltd., Canada) equipped with an 11.1 PBq source at 10 ± 0.5°C and operated at a dose rate of 10 kGy/h at the Advanced Radiation Technology Institute (ARTI) of the Korea Atomic Energy Institute (KAERI, Republic of Korea). The dosimetry was calibrated using 5-mm diameter alanine dosimeters (Bruker Instruments, Germany). Gamma-irradiated or non-irradiated mackerel meat samples were stored at 4°C for 20 days without a vacuum. TMA, VBN, color values, pH, ammonia nitrogen, and volatile organic compounds (VOCs) were measured at 10-day intervals.

### Measurement of TMA

The TMA content of gamma-irradiated or non-irradiated mackerel meat was determined according to a modified method by the Association of Official Analytical Chemists [28]. Twenty milliliters of 7.5%trichloroacetic acid (TCA) solution was added to 10 g of mackerel meat sample and then homogenized on the ice at 5,000 rpm for 1 min using a homogenizer (AM-7, Ace Homogenizer, Japan). Each sample was gently stirred for 30 min using a stirrer (MS-2026, Misung Scientific Co., Ltd., Korea), filtered (Advantec 5A, Japan), and then centrifuged at 1,977 ×*g* (Combi 514R, Hanil Science Industrial Co., Ltd., Korea) for 10 min. Then, 1 ml of 20%formaldehyde, 10 ml of anhydrous toluene, and 3 ml of saturated K_2_CO_3_ were sequentially added to 4 ml of the supernatant; this mixture was vortexed for 1 min and then allowed to stand for 5 min. The separated toluene supernatant was dehydrated using Na_2_SO_4_ for 1 min. The dehydrated supernatant was reacted with 0.02% picric acid at a ratio of 1:1, and optical absorbance was measured at 410 nm.

### Measurement of VBN

The VBN content of gamma-irradiated or non-irradiated mackerel meat was determined using a modification of Conway's method described in the Korean Food Standards Codex [29]. Fifty milliliters of distilled water were added to 10 g of mackerel meat sample, followed by vortexing and ultrasonic crushing for 2 min each, following which, samples were left at room temperature for 30 min. The mixtures were filtered, made up to 100 ml of distilled water, and used as sample solutions. Diffusion and titration reactions were performed by adding 1 ml of 0.01 NH_2_SO_4_ to the inner well, and 1 ml of each sample solution and saturated K_2_CO_3_ to the outer well of the Conway unit. The Conway unit was then sealed, shaken to uniformly mix the outer well solution, and incubated at 25°C for 1 h. Subsequently, 10 μl of Brunswick indicator was added to the inner well and titrated with 0.01 N NaOH using a micro-burette. Titration was repeated twice.

### Measurement of Color Values and pH

The color values of gamma-irradiated or non-irradiated mackerel meat were measured as lightness (L*), yellowness (b*), and redness (a*) using a colorimeter (A80F-208, Konica Minolta Inc., Japan). The measurements were repeated at least five times to obtain average values. The values of the standard color plate used were L* = 98.98, a* = 0.21, and b* = -0.28.

Gamma-irradiated or non-irradiated mackerel meat (3 g) was added to 30 mL distilled water and homogenized for 2 min at 10,000 rpm. The pH of the homogenized mackerel meat samples was measured at room temperature using a pH meter (*n* = 5; HM-25V, TOA, Japan).

### Measurement of Ammonia Nitrogen

The ammonia nitrogen content was measured according to the method described by Kim and Kim [27]. Gamma-irradiated or non-irradiated mackerel meat (10 g) was added to 100 ml of hot water and stirred for 10 min, followed by boiling in a water bath for 1 min. Subsequently, distilled water was added to the boiled meat to a final volume of 250 ml, mixed well, and filtered through filter paper (Advantec No. 2, Japan). To measure ammonia nitrogen levels, 0.1 ml of the filtrate and 2 ml each of solutions A (containing 0.005% sodium nitroprusside dehydrate and 1% phenol in distilled water) and B (containing 1% sodium hypochlorite, 0.6%sodium hydroxide, and 0.9% disodium hydrogen phosphate in distilled water) were mixed and reacted at 37°C for 20 min, and optical absorbance was measured at 630 nm.

### Measurement of Urease Activity

Urease activity was measured by quantifying the degree of color development after converting ammonia produced by the decomposition of urea, a substrate, to indophenol by reacting with phenol in the presence of hypochlorite [30]. UHEP buffer (200 μl, 20 mM HEPES buffer [N-2-hydroxyethylpiperazine-N'-2-ethane sulfonic acid], pH 7.5, containing 30 mM urea, 1 mM EDTA, 1 mM 2-mercaptoethanol) was added to 50 μl of crude urease and reacted at 37°C for 30 min. Thereafter, 400 μl of phenol nitroprusside and 400 μl of alkaline hypochlorite were added and reacted at 50°C for 10 min, and optical absorbance was measured at 625 nm. One unit (U) of urease activity was defined as the amount of urease required to hydrolyze 1 μM urea/min.

### Analysis of VOCs

The VOCs of gamma-irradiated or non-irradiated mackerel meat samples were analyzed under the conditions listed in Table 1 using an automatic thermal desorber (ATD650, USA) and a gas chromatography mass spectrometer (TQ8050, Shimadzu, Japan). The identities of the volatile compounds were matched with literature data (Willey/NBS Registry of Mass Spectra Data and Eight Peak Index of Mass Spectra) and the GCQ library search system (National Institute of Standards and Technology (NIST) mass spectra database). Compounds showing < 80% similarity and low peak values were classified as unknown substances.

### Statistical Analyses

Data are expressed as means ± SEM (*n* = 3). Statistical evaluation was performed by analysis of variance (ANOVA) using SAS software (ver. 9.4, SAS Institute, Inc., USA), according to Duncan's multiple range test (*p* < 0.05).

## Results and Discussion

### Effect of Gamma Irradiation on TMA Levels

In fish and shellfish, trimethylamine oxide (TMAO) is reduced to TMA by bacteria or enzymes after death, causing a fishy smell, and the TMA content determines the degree of spoilage [31, 32].

Changes in the amounts of TMA during storage at 4°C in gamma-irradiated or non-irradiated mackerel meat are reported in Table 2. The initial TMA content of whole mackerel samples was less than 1.58 mg/100 g, indicating the freshness of the initial product. During refrigeration of mackerel for 20 days, the TMA content was significantly increased with or without gamma irradiation. However, the increase in the TMA content of the gamma-irradiated groups was significantly lower compared to that of the control group (*p* < 0.05). On the 20th day, the TMA content of the non-irradiated group was 9.52 mg/100 g, indicating complete spoilage, and that of the 10 and 20 kGy irradiation groups was 2.23 and 1.69 mg/100 g, respectively, indicating that the fresh state was maintained.

These results were similar to those of a previous report evaluating the combined effect of gamma irradiation and refrigeration on the preservation of sea bream filets [33]. Moreover, Noh *et al*. [34] reported that TMA content increased as the storage period increased, and the rate of increase of the TMA content decreased as the gamma-irradiation dose increased. It was also reported that this result was closely related to microbial growth. According to the results of Noh and Kwon [35], TMA content did not differ from that of the non-irradiated group until 3 kGy, but reduced significantly at a higher dose, and this phenomenon was strongly related to microbial growth. These results suggested that the TMA content, according to the storage period, was affected by the irradiation dose, and the increased TMA content decreased as the irradiation dosage increased.

### Effect of Gamma Irradiation on VBN Levels

As fresh mackerel decays, the trace amounts of VBN present in the meat increase. As spoilage progresses, proteins are broken down into small molecules such as peptides, amino acids, and peptone, thereby increasing the VBN content [36]. In addition, the VBN content increases as TMAO is reduced by enzymes and microorganisms to produce basic substances such as TMA [37].

Table 3 shows the changes in the VBN content of mackerel meat after gamma irradiation during storage at 4°C. The initial VBN level of mackerel meat was 16.17 mg/100 g in the non-irradiated group and < 13.51 mg/100 g in the gamma-irradiated group. The VBN content of all samples increased with increasing storage period. However, the VBN content of each sample showed a significant difference after gamma irradiation. When stored for 20 days, the VBN contents of 0, 3, 7, 10, and 20 kGy gamma-irradiated samples were 33.39, 26.43, 25.43, 25.24, and 24.85 mg/100 g, respectively, indicating the lowest value when irradiated with 20 kGy.

According to a study by Chéour [38], the VBN content of sea bass irradiated with 0.5, 1, 2, and 3 kGy gamma irradiation on the 21st day of storage was lower than that of a non-irradiated group, suggesting that VBN content decreased as the irradiation dose increased. In addition, Noh and Kwon [35], who studied the quality of dried pollack during storage after irradiation, reported that VBN content significantly decreased as the irradiation dose increased, and explained that this was closely related to the growth of microorganisms in the sample.

### Effect of Gamma Irradiation on Color Values

To investigate the effect of gamma irradiation on the color values of mackerel meat, L*, a*, and b* color values were measured for each storage day (Table 4). On day 0 of storage, L*, a*, and b* values were all significantly increased by gamma irradiation. During the 20-day storage period, both the L* and b* values of the gamma-irradiated group and the non-irradiated group increased significantly compared to day 0. In 20 kGy irradiation, L* and b* values were observed to be 33.03 and 13.36, respectively. However, the a* values of the gamma-irradiated groups were significantly decreased compared to those on day 0, and the 20 kGy sample exhibited the lowest value of 9.03.

These results are similar to those of Riebroy *et al*. [39], who studied the storage stability after gamma irradiation of Som-fug, a fermented fish product produced from bigeye snapper. The L* and b* values of gamma-irradiated Som-fug samples were significantly increased during the 20-day cold storage period compared to the non-irradiated group. The increase in b* values during storage may be related to the formation of carbonyl compounds due to lipid or protein oxidation. These compounds can react with amino groups to form Maillard reaction products with a yellowish-brown color. The b* value of the gamma-irradiated sample was higher than that of the non-irradiated sample, which may be due to radiation-induced oxidation of myoglobin [39, 40]. According to the results of the study by Dvořák *et al*. [41], the a* value of *Oncorhynchus mykiss* irradiated with gamma rays decreased and the pinkish color was lost. This was consistent with our findings.

### Effect of Gamma Irradiation on pH Values

Changes to the pH of mackerel meat samples with and without gamma irradiation during cold storage are shown in Table 5. On day 0, the pH of the non-irradiated group was 5.86 and was significantly decreased by gamma irradiation. As the storage period increased, the pH was significantly increased in both the irradiated and non-irradiated groups. By day 20, the pH of the non-irradiated group was 7.46, which was at the level of complete decay, whereas those of the 3, 7, 10, and 20 kGy gamma-irradiated groups were 6.51, 5.99, 6.10, and 6.11, respectively, indicating that freshness was maintained when irradiated with 7, 10, and 20 kGy. These trends are similar to the TMA and VBN results.

The pH of fresh fish after death is approximately 5.5-6.5 [42], and it is known that freshness decreases over storage time and pH increases. Generally, when the pH of red meat fish is between approximately 6.2-6.4, initial spoilage is indicated, and a pH value of 6.5 or higher means that the fish is sufficiently rotten so as to be considered unfit for consumption [43]. An increase in the pH value indicates the accumulation of alkaline compounds such as TMA and ammonia compounds, mainly from microbial flora [44].

### Effect of Gamma Irradiation on Ammonia Nitrogen Levels

The changes in ammonia nitrogen content of samples with and without gamma irradiation during cold storage are shown in Table 6. The ammonia nitrogen content of the non-irradiated group on day 0 of storage was 0.11 mg/ 100 g, which was significantly reduced by irradiation with 7, 10, and 20 kGy. During the 20-day storage period, both the gamma-irradiated group and the non-irradiated group exhibited significantly increased ammonia nitrogen content, but the increase in the gamma-irradiated group was lower than that of the non-irradiated group. On day 10 of storage, the ammonia nitrogen content of the 10 and 20 kGy-irradiated groups was significantly decreased by approximately 10.3% compared to the non-irradiated group. On day 20 of storage, the 3, 7, 10, and 20 kGy gamma-irradiated groups had significantly decreased levels by around 6.5, 15.2, 17.4, and 23.9%, respectively, compared to the non-irradiated group.

Kim *et al*. [45] and Byun *et al*. [46] reported that the ammonia nitrogen content shows an overall tendency to increase as the storage period increases, but that it was suppressed as the gamma-irradiation dose increased. This could be because the growth of microorganisms, the main cause of ammonia nitrogen production, is being inhibited, or their physiological activity is reduced by irradiation with gamma rays. Therefore, it is possible to extend the shelf life of mackerel by gamma irradiation.

According to these results, gamma irradiation is thought to reduce off-flavor by delaying the spoilage of fish. The results also suggest the possibility that the action of urease is inhibited by reducing the proliferation of microorganisms.

### Effect of Gamma Irradiation on Urease Activity Inhibition

Table 7 shows the changes in the activity of the crude urease enzyme extracted from *V. parahaemolyticus* after gamma irradiation. The 0, 3, 7, 10, and 20 kGy gamma-irradiated groups showed urease activities of 26.12, 19.68, 17.97, 17.38, and 15.91 unit/ml, respectively. These results indicate that urease enzyme activity was significantly reduced in the gamma-irradiated group compared to that in the non-irradiated group. In general, the urea contained in fish is decomposed by urease secreted by microorganisms to produce ammonia. Fish smell occurs due to increased pH and the volatility of flesh due to the effect of ammonia [5, 47]. The results of this study showed that urease activity was inhibited by gamma irradiation, thereby suggesting the usefulness of gamma irradiation to suppress fishy smell by inhibiting urease activity.

### Effect of Gamma Irradiation on VOCs

Analysis of changes in VOC content of gamma-irradiated mackerel meat during storage for 20 days revealed a total of 37 VOCs, including 14 aldehydes, 6 alcohols, 4 ketones, and 13 other compounds (data not shown). Table 8 presents the changes in the contents of ethanol, 2-butanone, 3-methylbutanal, and trans-2-pentenal, which are known off-flavor components produced by fish spoilage, based on storage duration. On day 0, the contents of ethanol, 2-butanone, and 3-methybutanal in the groups irradiated with 3 kGy were decreased by 4.18, 4.44, and 5.04%, respectively, compared to the non-irradiated group. On the other hand, the content of trans-2-pentenal decreased depending on the intensity of gamma irradiation. On day 10 of storage, the ethanol content of the 3 and 7 kGy-irradiated groups decreased by 7.84 and 13.36%, respectively, compared to the non-irradiated group, and the 2-butanone content of the 3 kGy-irradiated group decreased by 2.97% compared with the non-irradiated group. The content of 3-methylbutanal and trans-2-pentenal in all gamma-irradiated groups was lower than that in the non-irradiated group. On day 20 of storage, the contents of ethanol, 2-butanone, 3-methylbutanal, and trans-2-pentenal in all gamma-irradiated groups were decreased compared to those in the non-irradiated group. In addition, the increased ethanol, 2-butanone, 3-methylbutanal, and trans-2-pentenal contents compared to day 0 were found to be lowest in the 10 kGy irradiation group, and decreased by 66.10, 48.49, 66.49, and 78.49%, respectively, compared to the non-irradiated group.

In a previous study concerning the characteristics of gamma-irradiated salted and fermented anchovy sauce, aldehydes, esters, ketones, and S-containing compounds were reduced by 10 kGy irradiation. This suggests that 10 kGy irradiation could induce radiolysis of several functional groups in compounds, including aldehydes, esters, ketones, S-containing compounds, and furans [48].

## Figures and Tables

**Table 1:** Operating conditions of GC/MS (Gas Chromatography Mass Spectrometry).

GC/MS (QP- 2010, Shimadzu, Japan)	
Oven temperature	35°C (10 min)
	8°C/ min – 120°C (10 min)
	12°C/ min – 180°C (7 min)
	15°C/ min – 230°C (10 min)

Column	At-1 60 m × 0.32 mm × 1.0 μm
Ion source temperature	200°C
Interface temperature	250°C
Solvent cut time	1.00 min
Detector gain mode	Relative to the tuning result
Detector gain	+ 0.00 kV
Threshold	0

**Table 2:** Changes in trimethylamine (TMA) levels of mackerel meats with or without gamma irradiation during storage at 4°C.

Storage period (days)	Treatments (kGy)				

	0	3	7	10	20
0	1.48 ± 0.10^ABc^	1.58 ± 0.04^Ab^	1.41 ± 0.02^ABCc^	1.30 ± 0.02^BCb^	1.19 ± 0.02^Cb^
10	6.02 ± 0.03^Ab^	4.42 ± 0.04^Bb^	3.11 ± 0.01^Cb^	2.19 ± 0.02^Da^	1.66 ± 0.01^Ea^
20	9.52 ± 0.08^Aa^	5.95 ± 0.04^Ba^	3.93 ± 0.02^Ca^	2.23 ± 0.05^Da^	1.69 ± 0.07^Ea^

The TMA levels are measured as mg/100 g. Means in columns (a-c) and rows (A-E) with different superscript letters are significantly different (*p* < 0.05).

**Table 3:** Changes in volatile basic nitrogen (VBN) values of mackerel meats with or without gamma irradiation during storage at 4°C.

Storage period (days)	Treatments (kGy)				

	0	3	7	10	20
0	16.17 ± 0.01^Ab^	10.92 ± 0.01^Ac^	12.50 ± 0.01^Ac^	12.57 ± 0.00^Ac^	13.51 ± 0.02^Ac^
10	29.26 ± 3.46^Aa^	19.74 ± 1.88^Bb^	19.25 ± 2.18^Bb^	18.97 ± 0.99^Bb^	18.34 ± 1.88^Bb^
20	33.39 ± 0.39^Aa^	26.43 ± 1.24^Ba^	25.43 ± 0.17^Ba^	25.24 ± 1.44^Ba^	24.85 ± 2.28^Ba^

The VBN levels are measured as mg/100 g. Means in columns (a-c) and rows (A-C) with different superscript letters are significantly different (*p* < 0.05).

**Table 4:** Changes in color values of mackerel meats with or without gamma irradiation during storage at 4°C.

Color values	Treatments (kGy)	Storage period (days)		

		0	10	20
L*	0	27.28 ± 0.60^Bb^	30.46 ± 1.20^Ab^	30.49 ± 1.64^Ac^
	3	28.83 ± 2.16^Ba^	29.99 ± 0.29^Bb^	31.67 ± 0.39^Ab^
	7	28.29 ± 0.55^Ca^	30.36 ± 0.44^Bb^	31.52 ± 0.60 ^Ab^
	10	28.52 ± 0.46^Ca^	30.65 ± 0.49^Bb^	31.95 ± 0.41^Ab^
	20	28.50 ± 0.74^Ba^	32.65 ± 1.17^Aa^	33.03 ± 0.31^Aa^
a*	0	13.67 ± 0.34^Bb^	14.14 ± 0.72^Aa^	13.38 ± 0.17^Ba^
	3	14.47 ± 0.40^Aa^	11.20 ± 0.36^Bb^	11.42 ± 0.14^Bb^
	7	14.39 ± 0.27^Aa^	11.05 ± 0.69^Bb^	9.51 ± 0.35^Cd^
	10	14.71 ± 0.41^Aa^	11.02 ± 1.17^Bb^	10.20 ± 0.15^Cc^
	20	14.51 ± 0.68^Aa^	9.27 ± 0.63^Bc^	9.03 ± 0.31^Be^
b*	0	7.24 ± 0.24^Cc^	8.92 ± 0.35^Bb^	9.03 ± 0.49^Ac^
	3	8.10 ± 1.18^Bb^	9.95 ± 0.32^Aa^	9.69 ± 0.19^Ac^
	7	7.92 ± 0.37^Bb^	9.31 ± 0.69^Aab^	10.59 ± 0.61^Ab^
	10	8.38 ± 0.80^Aab^	8.70 ± 1.53^Ab^	9.44 ± 0.84^Ac^
	20	8.87 ± 0.62^Ca^	9.96 ± 1.36^Ba^	13.36 ± 0.88^Aa^

Means in columns (a-c) and rows (A-C) with different superscript letters are significantly different (*p* < 0.05).

**Table 5:** Changes in pH of mackerel meats with or without gamma irradiation during storage at 4°C.

Storage period (days)	Treatments (kGy)				

	0	3	7	10	20
0	5.86 ± 0.18^BCb^	5.67 ± 0.01^Bb^	5.67 ± 0.02^Bb^	5.69 ± 0.00^Bb^	5.73 ± 0.01^Ca^
10	6.82 ± 0.21^Ba^	5.72 ± 0.03^Bb^	5.80 ± 0.01^ABb^	5.79 ± 0.02^Bb^	5.82 ± 0.01^Bb^
20	7.46 ± 0.12^Aa^	6.51 ± 0.04^Ab^	5.99 ± 0.07^Ac^	6.10 ± 0.07^Ac^	6.00 ± 0.04^Ac^

Means in columns (a-c) and rows (A-E) with different superscript letters are significantly different (*p* < 0.05).

**Table 6:** Changes in ammonia nitrogen content of mackerel meats with or without gamma irradiation during storage at 4°C.

Storage period (days)	Treatments (kGy)				

	0	3	7	10	20
0	0.11 ± 0.01^Ac^	0.11 ± 0.01^Ac^	0.09 ± 0.02^Bc^	0.07 ± 0.01^Cc^	0.06 ± 0.01^Cc^
10	0.29 ± 0.01^Ab^	0.28 ± 0.01^Ab^	0.28 ± 0.01^Ab^	0.26 ± 0.01^Bb^	0.26 ± 0.01^Bb^
20	0.46 ± 0.01^Aa^	0.43 ± 0.02^Ba^	0.39 ± 0.02^Ca^	0.38 ± 0.02^Ca^	0.35 ± 0.01^Da^

The ammonia nitrogen contents are measured as mg/100 g. Means in columns (a-c) and rows (A-D) with different superscript letters are significantly different (*p* < 0.05).

**Table 7:** Urease catalytic activity according to gamma irradiation level.

	Treatments (kGy)				

	0	3	7	10	20
Urease enzyme activity	26.12 ± 4.15^A^	19.68 ± 1.66^B^	17.97 ± 1.26^B^	17.38 ± 0.51^B^	15.91 ± 1.43^C^

Urease catalytic activities are measured as units/mg. Means in the same column (A-B) with different superscript letters are significantly different (*p* < 0.05).

**Table 8:** Changes in volatile organic compounds (VOCs) of mackerel meats with or without gamma irradiation during storage at 4°C.

Storage period (days)	Treatment (kGy)	VOCs (Area × 10^5^)			

		Ethanol	2-Butanone	3-Methylbutanal	Trans-2-pentenal
0	Control	214.64 ± 0.00	77.27 ± 0.00	33.52 ± 0.00	32.87 ± 0.00
	3	205.67 ± 22.38	73.84 ± 12.22	31.83 ± 2.04	23.23 ± 2.40
	7	241.15 ± 30.70	109.43 ± 41.49	45.34 ± 29.04	23.60 ± 8.25
	10	288.42 ± 3.06	119.36 ± 29.67	47.31 ± 16.48	22.10 ± 10.93
	20	322.73 ± 52.40	101.84 ± 13.67	44.32 ± 12.89	14.11 ± 0.41
10	Control	369.08 ± 9.96	59.86 ± 0.83	39.07 ± 1.07	15.63 ± 3.14
	3	340.16 ± 9.16	58.08 ± 3.46	18.32 ± 2.65	12.59 ± 1.81
	7	319.78 ± 10.59	68.89 ± 4.07	24.84 ± 0.22	13.65 ± 1.99
	10	420.31 ± 189.31	76.31 ± 5.97	24.16 ± 0.59	14.60 ± 0.87
	20	495.04 ± 78.07	81.08 ± 0.87	32.29 ± 2.05	13.60 ± 0.62
20	Control	842.73 ± 0.70	157.49 ± 43.20	114.79 ± 7.60	108.07 ± 38.71
	3	403.66 ± 17.78	121.23 ± 8.18	80.52 ± 4.95	35.50 ± 3.22
	7	421.37 ± 86.23	127.30 ± 3.14	61.60 ± 1.55	17.14 ± 1.19
	10	383.86 ± 0.44	125.31 ± 1.85	54.30 ± 3.87	15.63 ± 1.30
	20	517.11 ± 87.55	122.45 ± 0.28	58.54 ± 2.01	13.25 ± 0.30

Each value is the mean of duplicate measurements of pooled samples.
